# Mapping Cucumber Vein Yellowing Virus Resistance in Cucumber (*Cucumis sativus* L.) by Using BSA-seq Analysis

**DOI:** 10.3389/fpls.2019.01583

**Published:** 2019-12-03

**Authors:** Marta Pujol, Konstantinos G. Alexiou, Anne-Sophie Fontaine, Patricia Mayor, Manuel Miras, Torben Jahrmann, Jordi Garcia-Mas, Miguel A. Aranda

**Affiliations:** ^1^Centre for Research in Agricultural Genomics (CRAG) CSIC-IRTA-UAB-UB, Plant and Animal Genomics Program, Barcelona, Spain; ^2^Institut de Recerca i Tecnologia Agroalimentàries (IRTA), Genomics and Biotecnology Program, Barcelona, Spain; ^3^Semillas Fitó S.A., Biotechnology Department, Barcelona, Spain; ^4^Centro de Edafología y Biología Aplicada del Segura (CEBAS)-CSIC, Departamento de Biología del Estrés y Patología Vegetal, Murcia, Spain

**Keywords:** Cucumber vein yellowing virus, cucumber, resistance, mapping, BSA-seq, breeding, marker-assisted selection

## Abstract

Cucumber vein yellowing virus (CVYV) causes severe yield losses in cucurbit crops across Mediterranean countries. The control of this virus is based on cultural practices to prevent the presence of its vector (*Bemisia tabaci*) and breeding for natural resistance, which requires the identification of the loci involved and the development of molecular markers for linkage analysis. In this work, we mapped a monogenic locus for resistance to CVYV in cucumber by using a Bulked Segregant Analysis (BSA) strategy coupled with whole-genome resequencing. We phenotyped 135 F_3_ families from a segregating population between a susceptible pickling cucumber and a resistant Long Dutch type cucumber for CVYV resistance. Phenotypic analysis determined the monogenic and incomplete dominance inheritance of the resistance. We named the locus *CsCvy-1*. For mapping this locus, 15 resistant and 15 susceptible homozygous F_2_ individuals were selected for whole genome resequencing. By using a customized bioinformatics pipeline, we identified a unique region in chromosome 5 associated to resistance to CVYV, explaining more than 80% of the variability. The resequencing data provided us with additional SNP markers to decrease the interval of *CsCvy-1* to 625 kb, containing 24 annotated genes. Markers flanking *CsCvy-1* in a 5.3 cM interval were developed for marker-assisted selection (MAS) in breeding programs and will be useful for the identification of the target gene in future studies.

## Introduction

Cucumber vein yellowing virus (CVYV) is an ipomovirus (family *Potyviridae*) that is transmitted in a semi-persistent manner by the whitefly *Bemisia tabaci*. CVYV was first reported in Eastern countries of the Mediterranean basin (Israel, Jordan, Turkey, and Cyprus) later expanding to Western Mediterranean countries including Spain, Portugal, France, and Tunisia (reviewed in Navas-Castillo et al., 2014). CVYV infects cucurbits, causing symptoms of variable intensity. In melon (*Cucumis melo* L.) and cucumber (*Cucumis sativus* L.), it causes a typical severe vein clearing often followed by generalized chlorosis and necrosis. In cucurbit-producing areas of heavy *B. tabaci* infestation, it can cause epidemics with massive yield losses and dramatic economic consequences. Although significant diversity has been reported for this virus, epidemics in Western Mediterranean countries seem to be associated to genetically uniform virus populations (Desbiez et al., 2019), perhaps as a consequence of single virus introductions followed by rapid epidemic expansions (Janssen et al., 2007). At the start of the CVYV epidemics, disease control relied heavily on early detection (Martínez-García et al., 2004) and eradication, and whitefly control. Sources of resistance were soon identified in cucumber (e.g. Picó et al., 2003) and indeed, commercial seed companies are currently selling cucumber hybrids resistant to CVYV, which represent an excellent solution for disease control. Resistant accessions, varieties and hybrids seem to share the common characteristic that resistance is partial; symptoms in inoculated plants are mild or absent, and the virus can be detected infecting systemically the so-called resistant plants, although at reduced levels as compared to susceptible controls (*e.g.* Galipienso et al., 2013).

Breeding for CVYV resistance of cucumber varieties requires the development of molecular markers linked to the trait of interest, as pathology tests are labor-intensive and time-consuming. For molecular breeding, several genetic and genomics resources are available for cucumber, which have substantially increased after the release of reference genomes (Huang et al., 2009; Wóycicki et al., 2011; Yang et al., 2012). Cucumber has a relatively small genome (367 Mb, 2n = 2x = 14) with a very narrow genetic base (Staub et al., 2008; Wóycicki et al., 2011). The availability of high-density consensus maps and whole genome sequencing has facilitated the identification of NB-LRR resistance genes in the cucumber genome and the map-based cloning of candidate genes (Ren et al., 2009; Yang et al., 2013). These achievements are the basis for efficient marker-assisted selection (MAS). In this sense, Bulked Segregant Analysis (BSA) was developed as a rapid method for the detection of molecular markers linked to target traits in mapping populations (Michelmore et al., 1991). The principle of BSA is the selection of a small group of individuals from a segregating population that belong to phenotypic contrasting extremes of the target trait. These individuals are then pooled in two bulks, and fingerprinted to obtain genetic polymorphisms. When the trait is monogenic, the number of individuals per bulk can be reduced to 10-20, but in the case of quantitative trait loci (QTL) this number should be increased (Sun et al., 2010; Takagi et al., 2013; Zou et al., 2016). With the improvement of technologies and the significant reduction of next generation sequencing (NGS) costs, whole-genome resequencing has been coupled to BSA. The combination of BSA with NGS (BSA-seq) has accelerated the identification of tightly linked markers for important traits, improving the resolution of maps for gene identification and QTL mapping (Zou et al., 2016). In cucumber, BSA-seq has been successfully applied for mapping traits such as early flowering (Lu et al., 2014), flesh thickness (Xu et al., 2015) and downy mildew resistance (Win et al., 2017).

The aims of this work were to study the inheritance of the resistance conferred by the resistant accession CE0749 in a segregating F_2:3_ population, to map the *CsCvy-1* locus by using a BSA-seq approach, and to develop molecular markers that are easily transferable to cucumber breeding programs.

## Materials and Methods

### Plant Material and Phenotyping for CVYV Resistance

Accession CE0754 (hereafter P_S_), a CVYV-susceptible pickling cucumber, was crossed with accession CE0749 (hereafter P_R_), a CVYV-resistant Long Dutch type cucumber, to obtain the F_1_, F_2_ and F_2:3_ segregating populations used to perform the genetic mapping of the resistance trait. P_S_, P_R_, F_1_, F_2_, and F_2:3_ plants were inoculated mechanically with CVYV-AILM (Martínez-García et al., 2004) by rubbing recently-expanded cotyledons with extracts from CVYV-infected cucumber plants (cv. SMR-58) and were re-inoculated three days after. To measure virus accumulation in P_S_, P_R_ and F_1_-inoculated plants, we followed procedures described by Marco et al. (2003); quantitative dot-blot hybridization was done using the probe described by (Martínez-García et al., 2004). Plants were sampled at 9, 16, 23, and 30 days post inoculation (dpi) taking three leaf discs measuring 8 mm in diameter per leaf sampled. Symptoms were scored using a 0–3 scale: (0) No symptoms; (1) mild chlorotic mottling in young but fully-expanded leaves in interveinal petiole-proximal leaf areas; (2) similar to (1) plus vein yellowing evident in fully-expanded leaves and incipient in young developing leaves; (3) obvious vein yellowing in all leaves, including young developing leaves, chlorotic mosaics in fully-expanded leaves and overall plant growth reduction. A minimum of nine plants were used per treatment. Plants were kept in an insect-proof glasshouse, with temperature control set at 26°C/18°C (day/night) throughout the experiments.

### DNA Extraction and NGS Sequencing

Young leaves from the parental lines and the F_2_ population were collected, frozen in liquid nitrogen, and stored at −80°C. DNA was extracted following the CTAB method (Doyle, 1991), adding a purification step using Phenol : Chloroform:Isoamyl alcohol (25:24:1). The integrity of DNA was evaluated by agarose gel electrophoresis and quantified with the PicoGreen^®^ dsDNA Assay Kit (Life Technologies) according to the manufacturer’s protocol. For NGS sequencing, we pooled equimolar concentrations of DNA from 15 homozygous resistant F_2_ plants (R-Bulk), and from 15 homozygous susceptible F_2_ plants (S-Bulk). Twenty µg aliquots of each bulked DNA and both parental lines were sent to the National Centre for Genomic Analysis (CNAG-CRG, Barcelona, Spain) for library construction and sequencing. Libraries of 300 bp and 500 bp average insert size for bulks and parents, respectively, were sequenced with Illumina HiSeq 2000 (Illumina, Inc. San Diego, CA, USA), generating 2 × 100 bp paired-end reads for both datasets.

### Conventional Linkage Mapping

A set of 172 polymorphic cucumber SNP markers, distributed across the seven chromosomes of the cucumber genome, were selected between P_R_ and P_S_ from the resequencing data (**Supplementary Table 1**). Kompetitive Allele Specific PCR (KASP, www.lgcgroup.com) was used for genotyping a subset of 72 individuals, with the 172 SNPs converted to KASP markers, following the protocol of LGC Genomics. A genetic map was constructed using JoinMap^®^ 5 (Kyazma, B.V.), with 172 SNP markers data and the phenotypic data used as another marker (*CsCvy-1*) due to the monogenic inheritance of the trait. Three F_2_ individuals and one SNP marker were excluded because of the high amount of missing data. Two more SNP markers were excluded for being identical (similarity value = 1.000) to other nearby markers. Linkage analysis and marker order were performed with the regression mapping algorithm, and genetic distance was calculated using the Kosambi mapping function (Crow, 1990). QTL analysis was performed using MapQTL6^®^ (Kyazma B.V.) using both interval mapping and Kruskal-Wallis (KW) analysis.

### Variant Detection and BSA-seq Analysis

#### Variant Detection and Functional Effect Annotation

In order to detect variants of the genome linked to the resistance against CVYV, a BSA-seq strategy was implemented in which two pools of F_2_ individuals were chosen depending on their phenotype. 15 homozygous resistant F_2_ individuals (R-bulk) and 15 homozygous susceptible F_2_ individuals (S-bulk) were selected for pooling and sequencing. Paired-end Illumina sequencing data from parental lines and the two bulks were trimmed (length ≥35 bp, with a mean sliding window of 4 bp phred quality score ≥20) using Trimmomatic (Bolger et al., 2014) and the output was quality checked using FastQC (www.bioinformatics.babraham.ac.uk/projects/fastqc/). Trimmed data were aligned versus the ChineseLong 9930 v3 assembly ftp://cucurbitgenomics.org/pub/cucurbit/genome/cucumber/Chinese_long/v3/) using the BWA-MEM algorithm (v0.7.16a-r1181; http://bio-bwa.sourceforge.net/bwa.shtml) with default parameters. After removal of unmapped reads and marking of PCR duplicates, variant calling was performed with samtools (v1.5; Li, 2011) using default parameters, except for the following: mapping quality ≥10 and base quality ≥20. Variant calling format (VCF) files were filtered by applying the following criteria: genotype quality ≥10, depth ≥10, biallelic sites, alternative allele frequency ≤ 0.9, no missing data.

Structural variant (SV) analysis between the two parents was conducted using DELLY (Rausch et al., 2012) and Pindel (Ye et al., 2009). Both programs were run with default parameters. Raw data underwent technical- and visual-based filtering. The technical filters applied were the following: read depth ≥10 in at least one sample, parents variable between them, variant size larger than 50 bp and smaller than 50,000 bp. Remained variants were visually inspected in IGV (Robinson et al., 2011) to avoid cases of false positives.

Annotation of the functional effect of the variants was done using snpEff (version 4.3t; (Cingolani et al., 2012).

#### BSA-seq Analysis

BSA-seq analysis was performed using the R package QTLseqr (Mansfeld and Grumet, 2018). More specifically, SNPs for the 2 bulks were first filtered using the function “filterSNPs”, by keeping positions with 30 ≤ total depth ≤ 150, 0.2 ≤ reference allele frequency ≤0.8 and genotype quality ≥30. For the QTL detection, the (Takagi et al., 2013) method was applied, implemented by the function “runQTLseqAnalysis”. Briefly, SNP-index was calculated for both S- and R-bulk by dividing the number of non-reference alleles with the total number of reads in a position. If the SNP-index was <0.3 in both bulks, the SNP was discarded. SNP-index values were calculated in sliding windows of 4.7 Mbp with a 10 kb step and the average SNP-index value for each window was recorded along the chromosome. In order to avoid regions that generate segregation distortion caused by other reasons other than artificial selection (Takagi et al., 2013), the SNP-index of the S-bulk was subtracted by the SNP-index of the R-bulk in order to obtain the Δ(SNP-index). If Δ(SNP-index) equaled to 1.0 then the allele originated from the R parent and if Δ(SNP-index) equaled to -1 the allele originated from the S parent. Confidence interval calculations in each SNP position (95% and 99%), considering the null hypothesis (H_o_: no QTL), was done through a simulation analysis of 10,000 replications for two bulks that were randomly generated from the population and calculating, after each iteration, the SNP-index and the corresponding Δ(SNP-index) in the two simulated bulks.

## Results

### Phenotyping Analyses

**Figure 1:**
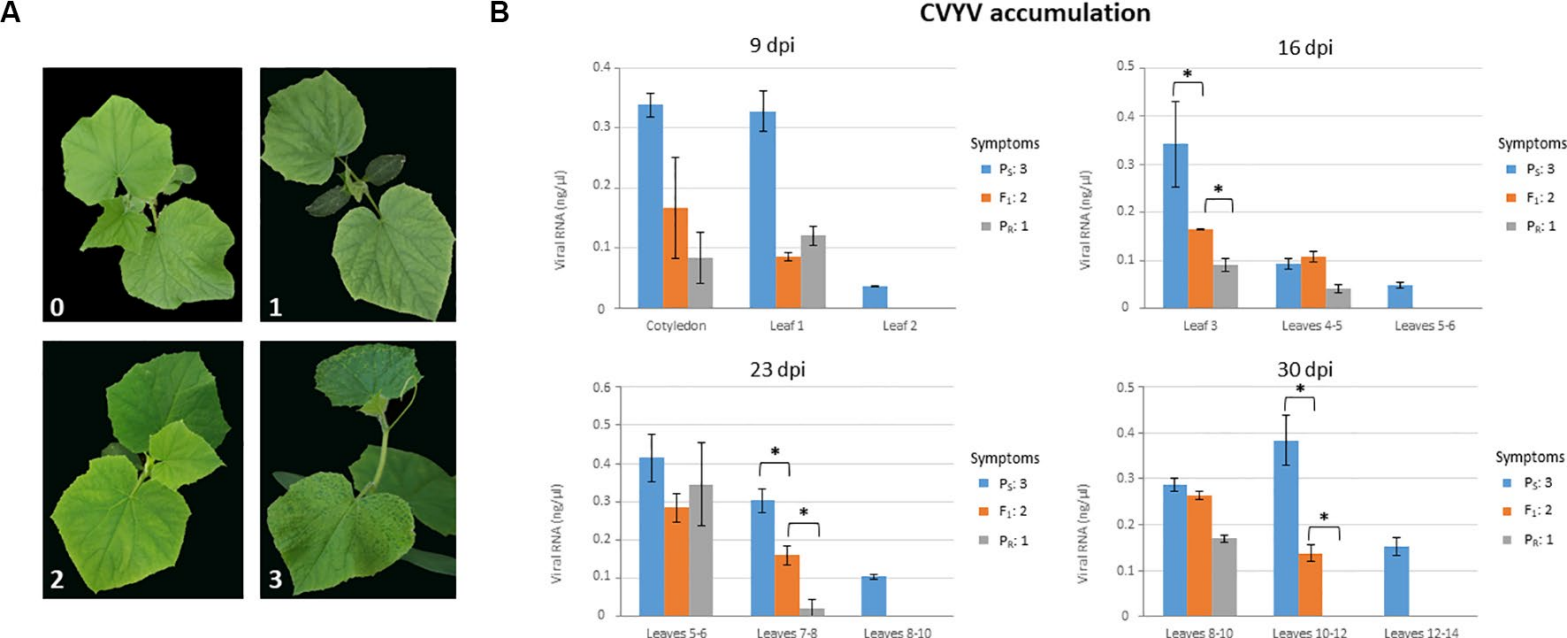
Phenotyping plants of the susceptible (P_S_) and resistant (P_R_) parental lines, and their F_1_, for CVYV susceptibility. Symptoms were scored and virus accumulation was measured at 9, 16, 23 and 30 days post-inoculation (dpi) **(A)** Symptoms in plants at 9 dpi. Symptoms could be assigned unequivocally to one of the following categories: (0) No symptoms; (1) mild chlorotic mottling in young but fully expanded leaves in interveinal petiole-proximal leaf areas; (2) similar to (1) plus vein yellowing evident in fully expanded leaves and incipient in young developing leaves; (3) obvious vein yellowing in all leaves, including young developing leaves, chlorotic mosaics in fully expanded leaves and overall plant growth reduction. **(B)** Virus accumulation was measured by quantitative dot-blot hybridization on total plant RNA extracts from basal, intermediate and apical leaves, as indicated for each graph. Three leaf discs (8 mm) were taken per leaf sampled. Virus accumulation in F_1_ plants was intermediate between the susceptible and resistant parental lines in basal and intermediate leaves at 16, 23 and 30 dpi, respectively; an asterisk marks statistically significant differences in Kruskal-Wallis tests (*P* < 0.027). A minimum of 9 plants were used per treatment. Symptom category (as in (A)) is indicated for each line on the right side of each graph for each time period after inoculation.

We first analyzed symptom development and virus accumulation in P_S_, P_R_, and F_1_ plants in a time course experiment (**Figure 1**). Symptoms started to appear in inoculated P_S_ plants as soon as 6 dpi (data not shown) and were conspicuous by 9 dpi, with plants showing obvious vein yellowing in all leaves and chlorotic mosaic in fully-expanded leaves (**Figure 1A**). Symptom display was delayed, and symptoms were clearly milder in P_R_-infected plants, although by 9 dpi all the plants showed mild interveinal chlorotic mottling in petiole-proximal leaf areas of fully expanded leaves (**Figure 1A**). Symptoms in F_1_ plants appeared at around 7 dpi, and by 9 dpi they were of intermediate severity between those of P_S_- and P_R_-infected plants; fully-expanded leaves showed chlorotic mottling but also mild vein yellowing, which was starting in young developing leaves (**Figure 1A**). The uniformity of symptoms in plants from each accession was remarkable, and thus each accession could be assigned to a symptom severity class without uncertainty for each observation time-point. Plants in this experiment were sampled and virus accumulation was measured, differentiating among samples from basal, medium and apical leaves (**Figure 1B**). Leaves from P_R_-infected plants accumulated significantly less virus than P_S_-infected leaves at all time points, except at 23 dpi in basal leaves, where no significant differences were found between both accessions. For F_1_-infected plants, differences in virus accumulation in leaves were less consistent, with an apparent trend suggesting intermediate levels of accumulation between those of P_R_ and P_S_; statistically significant differences (*P* < 0.027; Kruskal-Wallis tests) were found among the three accessions at 16 dpi in basal leaves and at 23 and 30 dpi in intermediate leaves (**Figure 1B**). Thus, virus accumulation data was essentially consistent with data on symptom expression, and phenotyping of F_1_ individuals suggested incomplete dominance of the resistance trait. In any case, symptom scoring at 9 dpi appeared to be robust enough to discriminate between susceptible and resistant plants.

To determine the resistance genotype of F_2_ individuals, 12 individuals from each of 137 F_2:3_ families were inoculated and symptom scoring performed at 9 dpi (**Supplementary Figure 1**). Out of these, 135 families were unequivocally assigned to susceptible, resistant or segregating for resistance phenotypes and, therefore, the 135 F_2_ individuals could be classified as: 37 homozygous for resistance, 36 homozygous for susceptibility and 62 heterozygous, which fit well (χ^2^ value 0.63, *P* > 0.05) with a 1:2:1 segregation ratio. We propose the symbol *CsCvy-1* for this monogenic incompletely-dominant resistance gene. For the BSA-seq analysis, two pools containing 15 F_2_ individuals homozygous for resistance and susceptibility to CVYV, respectively, were used.

### Preliminary F_2_ Mapping

**Figure 2:**
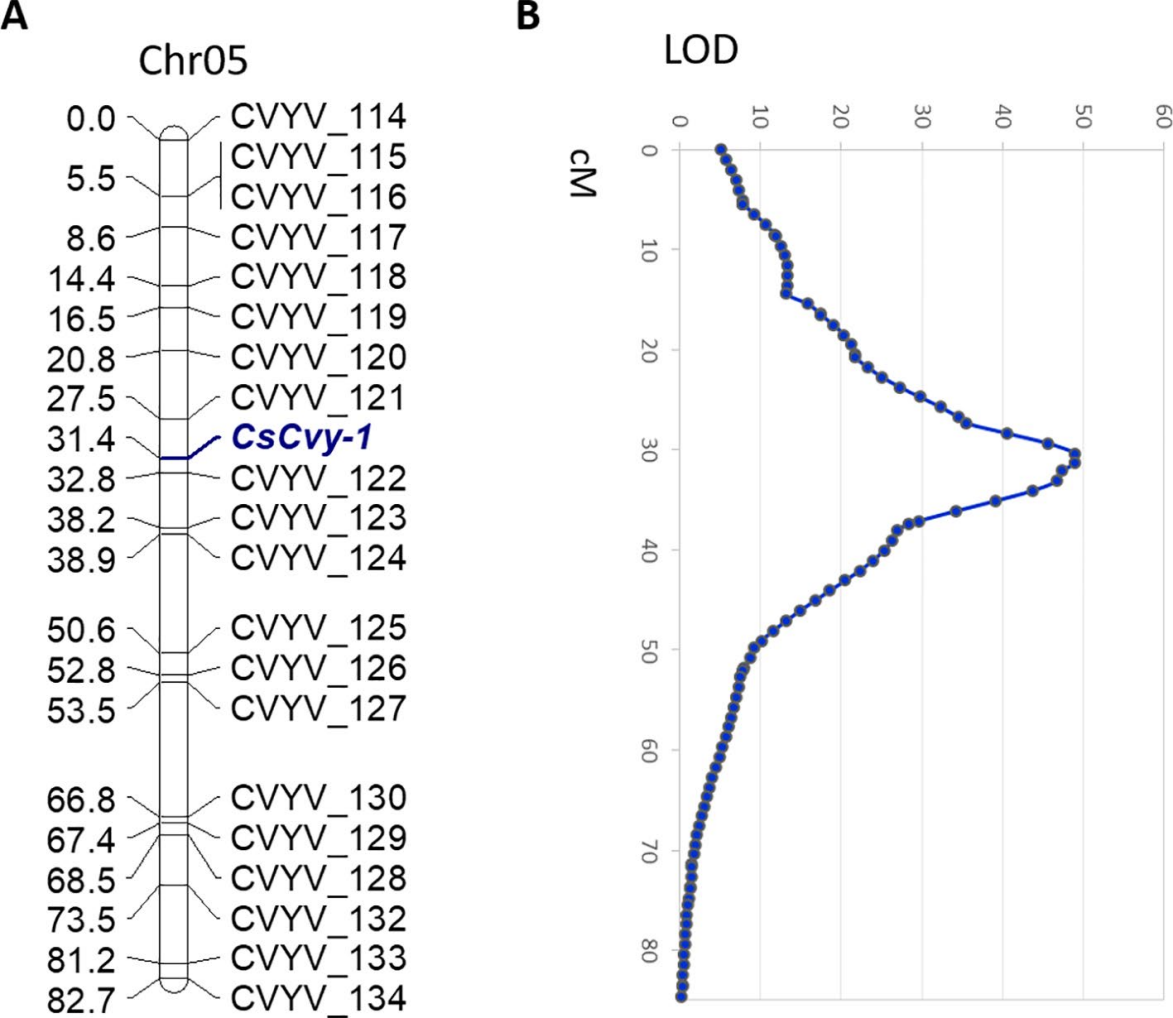
**(A)** Genetic linkage of SNP markers and the resistance locus *CsCvy-1* in Chr05. Genetic distance is expressed as centiMorgans (cM). The *CsCvy-1* gene is flanked by two markers in an interval of 5.3 cM. **(B)** Major QTL detected on Chr05 corresponding to *CsCvy-1* locus.

In parallel with the BSA-seq method, we performed F_2_ mapping with a subset of the population including the *CsCvy-1* locus as a phenotypic marker. We used 172 polymorphic SNPs covering the cucumber genome: 24 SNPs in Chr01, 18 SNPs in Chr02, 34 SNPs in Chr03, 36 SNPs in Chr04, 21 SNPs in Chr05, 27 SNPs in Chr06, and 12 SNPs in Chr07 (**Supplementary Table 1**). We selected 72 F_2_ individuals (35 homozygous resistant, 34 homozygous susceptible, 2 heterozygous, and 1 individual without phenotypic data) for mapping. The 72 F_2_ individuals were genotyped, and most of the markers fitted the expected 1:2:1 segregation ratio. However, a group of markers on Chr05 showed a distorted segregation. This distortion was also found for the phenotypic marker *CsCvy-1*, due to the selection of individuals, with almost all of them being homozygous for this trait. The genetic map consisted of eight linkage groups (LGs) spanning 628 cM, with Chr03 split into two linkage groups (LG3A, LG3B) (**Supplementary Figure 2**, **Supplementary Table 2**). The average marker interval was 3.9 cM, with a maximum distance of 23.6 cM. The longest LG was LG4, with 115.1 cM, and the shortest was LG3A with 14 cM. The *CsCvy-1* locus was mapped onto LG05, flanked by two markers, CVYV_121 and CVYV_122 in an interval of 5.3 cM (**Figure 2A**).

In order to discard any other minor QTL involved in the resistance trait, a QTL analysis was performed. As expected, we obtained a single major QTL on LG05, co-localizing with *CsCvy*-1, with a LOD value of 49 explaining 95.8% of the variance (**Figure 2B**). No other significant QTLs were observed, in accordance to the monogenic inheritance of the trait.

### Identification of *CsCvy-1* Locus by BSA-seq

Libraries of parental lines (P_S_ and P_R_), S-bulk and R-bulk were resequenced with the Illumina HiSeq2000 sequencer. In total, 274,709,874 paired-end clean reads were mapped, after trimming and adapter removal (**Table 1**).

**Table 1:** Results from the resequencing of P_S_, P_R_, S-bulk and R-bulk.

Samples	Clean mapped reads^1^	Mapping rate (%)^2^	Median depth	Coverage (%)^3^
**P**_S_	56,694,486	96	20 X	89
**P**_R_	60,357,172	96	22 X	89
**S-bulk**	77,718,714	95	30 X	90
**R-bulk**	79,939,502	96	31 X	90

^1^Number of reads after trimming and adapter removal.

^2^Alignment to the Chinese Long 9930 genome assembly v3 (www.cucurbitgenomics.org)

^3^Coverage (≥ 1 read).

**Figure 3:**
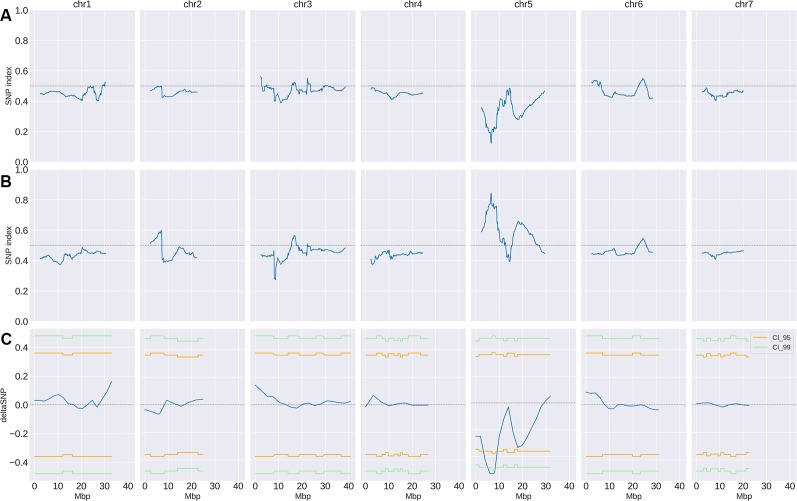
SNP-index and Δ(SNP-index) distribution (blue line). SNP-index for R-bulk **(A)** and S-bulk **(B)** was calculated using sliding windows of 4.7 Mbp in length with a step measuring 10 kb. The corresponding Δ(SNP-index) **(C)** was calculated as the difference SNP-index R-bulk – SNP-index S-bulk. Regions of statistical significance are detected as those that surpass the threshold of 0.95 (orange line) or 0.99 (green line) confidence intervals.

**Figure 4:**
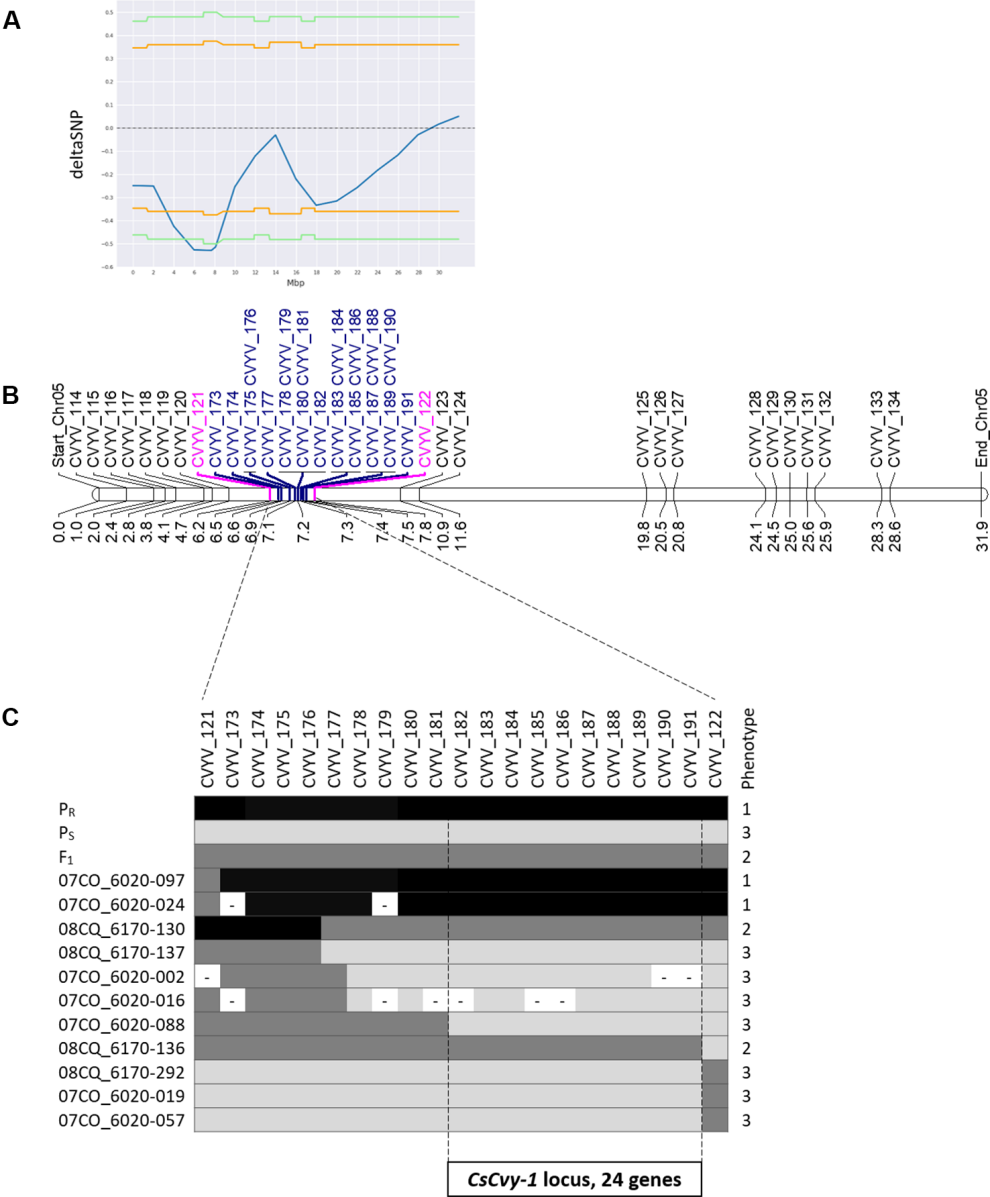
Scheme of fine mapping of the *CsCvy-1* locus. **(A)** DeltaSNP graph on Chr05 with the statistical confidence interval of 0.95 (orange line) or 0.99 (green line) **(B)** Physical map of Chr05 with all the markers used for mapping the *CsCvy-1* locus: preliminary mapping (black), flanking markers (magenta), fine mapping markers (blue). Distance is expressed in Mb on the bottom of the bar. **(C)** Genotyping result of recombinant individuals with the markers covering *CsCvy-1* locus. Alleles are represented in three colors: black for homozygous P_R_ allele, dark grey for heterozygous P_R_/P_S_, and light grey for homozygous P_S_ allele.

Small variant calling for the 4 datasets and subsequent variant filtering (see Material and Methods), generated 186,433; 215,735; 933,846 and 915,524 variants (SNPs and INDELs) for P_S_, P_R_, S-bulk and R-bulk, respectively, uniformly distributed throughout the genome (**Supplementary Table 3**). SNP variants from the 2 bulk datasets were used as input data in the QTLseqr R package for calculating SNP-index and Δ(SNP-index) for R- and S-bulks, based on the Takagi et al., 2013 method. Graphs for SNP-index of the R- and S-bulks and the Δ(SNP-index) were plotted (**Figure 3**). S-bulk was used as a reference dataset for the calculation of the Δ(SNP-index). BSA-seq analysis detected a single genomic region of 2,998,622 bp located in Chr05:5,088,092-8,208,448 bp, at a confidence interval higher than 0.99, associated with the *CsCvy-1* locus. The highest Δ(SNP-index) value was -0.528 at position 7,678,525 bp whereas the average Δ(SNP-index) was found to be -0.523 (**Figure 4A**). This region contained the flanking markers selected in the preliminary mapping.

### Fine Mapping of *CsCvy-1* Locus

In order to narrow down the interval of the *CsCvy-1* locus, fine mapping was performed using the linked SNPs obtained by BSA-seq on Chr05 to design 19 SNP markers (**Figure 4B**, **Supplementary Table 1**). A subset of the F_2_ population was genotyped to identify plants with recombination events, by using the genotyping data of the preliminary mapping. Eleven recombinant informative plants delimited the candidate area of *CsCvy-1* to a 626.5 kb interval, between the markers CVYV_181 and CVYV_122 (**Figure 4C**). This interval contains 24 annotated genes (**Table 2**). To further explore the genomic region containing the *CsCvy-1* locus, we extended our previous small variant analysis with the detection of SVs between the parental lines. In total, we detected 2 SVs of more than 50 bp in length: one 55 bp deletion and a duplication measuring 41,644 bp (**Supplementary Table 4**).

**Table 2:** List of genes within the interval of *CsCVY-1* locus in the Chinese Long 9930 v3 cucumber genome annotation, variants and structural variation analysis between P_S_ and P_R_.

Gene_ID	Description Chinese Long v3	Small variants^1^
CsaV3_5G011170*	Unknown protein	–
CsaV3_5G011180*	Serine/arginine repetitive matrix protein 2-like isoform X1	INDEL
CsaV3_5G011190*	Unknown protein	–
CsaV3_5G011200*	RNA-dependent RNA polymerase	SNP
CsaV3_5G011210*	RNA-dependent RNA polymerase	SNP
CsaV3_5G011220*	Endo-1,4-beta-xylanase A-like	SNP
CsaV3_5G011230	Unknown protein	SNP
CsaV3_5G011240	SAM dependent carboxyl methyltransferase	SNP
CsaV3_5G011250	Unknown protein	SNP
CsaV3_5G011260	SAM dependent carboxyl methyltransferase	SNP
CsaV3_5G011270	Unknown protein	–
CsaV3_5G011280	Integrator complex subunit 9 like	SNP
CsaV3_5G011290	Cytochrome c	–
CsaV3_5G011300	Serine/threonine protein kinase	SNP
CsaV3_5G012300	40S ribosomal protein S6	–
CsaV3_5G012310	Myb family transcription factor	–
CsaV3_5G012320	Protein kinase, putative	–
CsaV3_5G012330	E3 ubiquitin-protein ligase ORTHRUS 2-like	–
CsaV3_5G012340	THUMP domain-containing protein 1	–
CsaV3_5G012350	Amino acid transporter	–
CsaV3_5G012360	Unknown protein	–
CsaV3_5G012370	Perakine reductase	–
CsaV3_5G012380	UBN2_3 domain-containing protein	–
CsaV3_5G012390	SWI/SNF-related matrix-associated actin-dependent regulator of chromatin subfamily A containing DEAD/H box 1	–

^1^Small variants are referred as SNPs or INDELs < 50 bp.

*These genes are inside the duplicated region of 41,644 bp in P_R_.

With the purpose of detecting alterations in potential candidate genes that could be responsible for the resistance phenotype, we performed an annotation of the effect of small variations in the 24 genes found in the *CsCyv-1* region. In total, we annotated 45 SNPs/INDELs, of which 17 were highlighted for being within or close to coding sequences of 10 genes (**Supplementary Table 5**). The vast majority of variations (14 out of 17) were non-synonymous, and were annotated as modifiers or with low impact. Among the remaining four variants, three SNPs caused synonymous changes, and one insertion of 2 nucleotides caused a frameshift resulting in a premature stop codon (**Supplementary Table 5**). The three missense variants (moderate impact) were located in the coding sequence of CsaV3_5G011200, CsaV3_5G011220 and CsaV3_5G011240 genes, whereas the frameshift variant (high impact) was located in CsaV3_5G11180 gene. The frameshift caused the disruption of the CsaV3_5G11180 gene, coding for a serine/arginine repetitive matrix protein (SARMP) 2-like, at position 607 (**Supplementary Figure 3**). A search for conserved motifs detected a Constitutive Photomorphogenic 1 (COP1)-interacting protein signature. With regards to structural variations, we also detected a single duplication event in P_R_ measuring 41,644 bp in length located in Chr05:7,195,565-7,237,209 that contained genes CsaV3_5G011170 to CsaV3_5G011220 (**Table 2** and **Supplementary Figure 4**). Genes CsaV3_5G011170 and CsaV3_5G011190 encode for unknown proteins, CsaV3_5G11180 encodes the above-mentioned SARMP and genes CsaV3_5G011200 and CsaV3_5G011210 encode two RNA-dependent RNA polymerases (RDRs) 1a and 1b, respectively. In contrast, the deletion of 55 bp was located in an intergenic region.

## Discussion

In cucumber, the disease caused by CVYV is a limiting factor for production in areas with high pressure from viruliferous whiteflies. In this work, we phenotyped an F_2:3_ segregating population identifying a single monogenic locus, *CsCvy-1*, that controls resistance to CVYV. The resistance conferred by this locus is partial, as P_R_ plants showed viral accumulation in systemically infected leaves. However, the progression of the disease was very much reduced as compared to susceptible controls, the growth of the plants was not affected at all, and viral accumulation was significantly reduced as compared to P_S_ and F_1_ plants. In cucumber, the accession C.sat-10 (Picó et al., 2003) was described as having partial resistance against CVYV, and a segregating F_2_ population was obtained to study the genetic control of this resistance (Picó et al., 2008); the segregation fitted a monogenic control with dominance. These features are very similar to what we have described here; however, in the case of C.sat-10, no mapping studies were performed to determine the localization of the locus in the cucumber genome. Thus, although our resistance data fit well with those from Picó et al. (2003; 2008), we cannot rule out that both resistances to CVYV characterized in cucumber are independent. By comparing with closely related species such as melon, Pitrat et al. (2012) evaluated a collection of 1,188 accessions for resistance against CVYV, and studied the inheritance in F_1_, F_2_ and BC progenies. Three loci were detected in their work: *Cvy-1*, controlling resistance in PI 164323 and necrosis in HSD 93-20-A; *cvy-2*, showing recessive tolerance in HSD 2458, and *Cvy-3*, showing dominance for severe mosaic symptoms in Ouzbèque 2.

Breeding for resistance requires the availability of highly linked markers that can be utilized for performing rapid and specific introgressions of the desired trait. For marker development, conventional gene mapping is based on the phenotyping and genotyping of a large number of individuals in a population, and it is time-consuming, costly and limiting in terms of the size of the population (Takagi et al., 2013). One way to improve conventional mapping is to use BSA-seq, in which the number of individuals to be analyzed can be narrowed down to two representative bulks, and at the same time as the mapping, the resequencing data offers the possibility of obtaining a high number of markers linked to the trait (Yang et al., 2013). BSA-seq has been successfully applied for the mapping of important agronomical traits in many crops such as rice (Abe et al., 2012; Takagi et al., 2013; Yang et al., 2013; Sun et al., 2018), lettuce (Huo et al., 2016), potato (Kaminski et al., 2016), soybean (Song et al., 2017), broccoli (Shu et al., 2018; Branham and Farnham, 2019) or sorghum (Han et al., 2015). Moreover, in cucurbits BSA-seq has enabled the identification of candidate genes for dwarfism (Dong et al., 2018), yellow skin (Dou et al., 2018) and light rind color (Oren et al., 2019) in watermelon; mapping flavor traits in melon (Zhang et al., 2016); or the identification of candidate genes for flesh thickness (Xu et al., 2015), aphid resistance (Liang et al., 2016), early flowering QTL (Lu et al., 2014), two major QTLs for downy mildew resistance (Win et al., 2017), and three major QTLs conferring subgynoecy (Win et al., 2019) in cucumber. In the present study, through the use of a BSA-seq strategy, the *CsCvy-1* locus has been successfully mapped to a region of 2.9 Mb in chromosome 5, whereas no other regions of the genome exhibited significant association with the resistance. These results confirmed previous analysis performed with the conventional mapping approach and were later used for fine mapping. The BSA-seq analysis provided enough SNPs to fine map the trait within a narrow interval of 625 kb, containing only 24 annotated genes.

In order to identify candidate genes for *CsCvy-1*, we performed an analysis of small variants and structural variation around this locus. Most of the variations had a small, if any, predicted impact, except for the insertion of 2 nucleotides in the gene encoding a SARMP2-like, which causes a frameshift mutation in the coding sequence with the subsequent truncation of the protein. A functional analysis of the SARMP-like protein sequence detected a COP1-interacting protein signature. Interestingly, COP1 has been associated with plant pathogen resistance (Lim et al., 2018) and the *Arabidopsis thaliana* COP1-interacting protein is a positive regulator of ABA response (Ren et al., 2016), which is an essential regulator of plant immunity (Berens et al., 2017). Nevertheless, perhaps the most appealing modification within the *CsCvy-1* region is the duplication of the 41 Kb fragment containing the genes encoding RDRs 1a and 1b (CsaV3_5G011200 and CsaV3_5G011210). RDRs are critical players in RNA silencing pathways; they are the key enzymes in the process of amplification of double-stranded RNAs that activate gene silencing after nuclease processing. A role for RDRs in antiviral immunity has long been acknowledged, in particular for members of the RDR1 and RDR6 clades (Willmann et al., 2012). For instance, the absence of a functional RDR1 in *Nicotiana benthamiana* can explain enhanced susceptibility to many viruses in this species (Yang et al., 2004). In relation to virus resistance in crop species, it has been recently demonstrated that the tomato genes *Ty-1* and *Ty-3* for resistance to tomato yellow leaf curl virus are alleles of an RDR gene (Verlaan et al., 2013). In cucumber, a recent report shows that the RDR1a and 1b genes have enhanced expression in natural or engineered lines showing broad virus resistance; importantly, one of the viruses tested was CVYV (Leibman et al., 2018). Taking these data together, a mechanistic explanation for our observations may consist of enhanced antiviral activity in the P_R_ line as a consequence of enhanced RDR1a and/or 1b expression; this, in turn, would be the consequence of the described gene duplication. This is an attractive hypothesis that awaits further testing, although at least a good alternative candidate gene (*i.e.*, the gene encoding a SARMP2-like protein) exists. From the point of view of resistance stability when confronted to different CVYV strains (Desbiez et al., 2019), the potential implication of RDR1a/b represents an optimistic perspective, given its broad spectrum of action (Leibman et al., 2018).

In conclusion, in this work we identified the monogenic locus *CsCvy-1*, inherited under incomplete dominance, in a short interval of 5.3 cM containing 24 genes. This is the first report where a CVYV resistance locus has been mapped in cucumber, and valuable molecular markers for MAS breeding programs have been developed. Moreover, our findings will be the basis for further map-based cloning and functional validation of the resistance gene.

## Data Availability Statement

The raw data supporting the conclusions of this manuscript will be made available by the authors, without undue reservation, to any qualified researcher. Variation data were deposited to European Variation Archive (EVA: https://www.ebi.ac.uk/eva) with the project accession number PRJEB34274.

## Author Contributions

MA, JG-M, TJ, and MP conceived and designed the research. PM, MM and MA provided the plant material and performed the tests with CVYV. A-SF conducted the conventional mapping. KA performed the bioinformatics analysis of the BSA-seq. MP conducted marker development, mapping analysis, and wrote the manuscript with important contributions from MA and KA. All authors read and approved the final manuscript.

## Conflict of Interest

Authors A-SF and TJ were employed by company Semillas Fitó S.A.

The remaining authors declare that the research was conducted in the absence of any commercial or financial relationships that could be construed as a potential conflict of interest.
