# Supplementary material for: Mapping Cucumber Vein Yellowing Virus Resistance in Cucumber (Cucumis sativus L.) by Using BSA-seq Analysis
Source: Front Plant Sci. 2019 Dec 3;10:1583. doi: 10.3389/fpls.2019.01583 (PMC6901629; doi:10.3389/fpls.2019.01583)
Supplement: Supplementary file 1 [file Presentation_1.zip › Supplementary figure 2.pptx]

## Slide 1
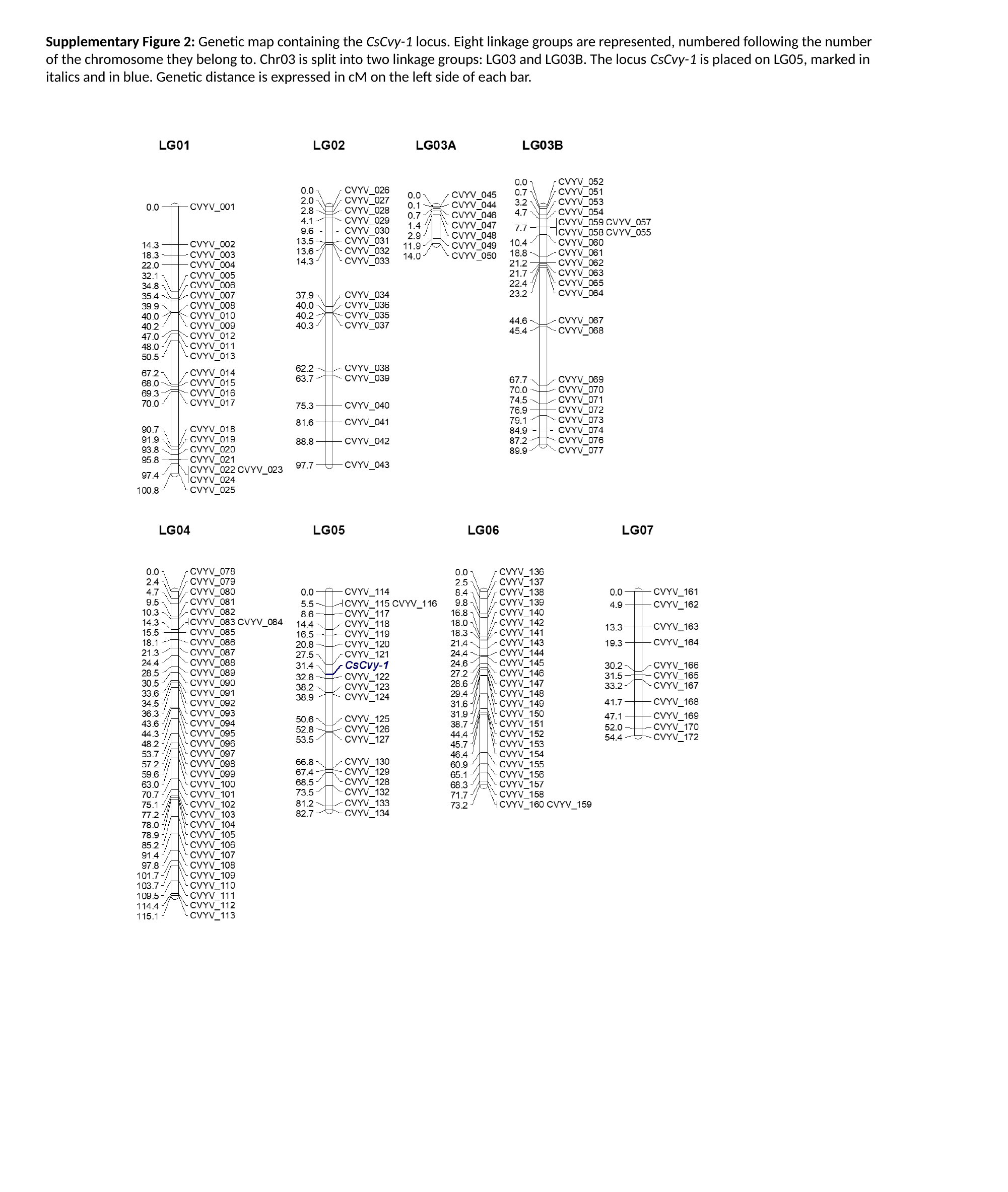

Supplementary Figure 2: Genetic map containing the CsCvy-1 locus. Eight linkage groups are represented, numbered following the number of the chromosome they belong to. Chr03 is split into two linkage groups: LG03 and LG03B. The locus CsCvy-1 is placed on LG05, marked in italics and in blue. Genetic distance is expressed in cM on the left side of each bar.
