# Supplementary material for: Mapping Cucumber Vein Yellowing Virus Resistance in Cucumber (Cucumis sativus L.) by Using BSA-seq Analysis
Source: Front Plant Sci. 2019 Dec 3;10:1583. doi: 10.3389/fpls.2019.01583 (PMC6901629; doi:10.3389/fpls.2019.01583)
Supplement: Supplementary file 1 [file Presentation_1.zip › Supplementary figure 3.PDF]

Supplementary Figure 3

```
CLUSTAL O(1.2.4) multiple sequence alignment


CsaV3_5G011180_wt              MSGGVADDGALDYARIQMIPSENRYEAFVCYGNEVDGLAEGNLDTLLLHLPELQELNSKG     60
CsaV3_5G011180_TGC->TGCGC      MSGGVADDGALDYARIQMIPSENRYEAFVCYGNEVDGLAEGNLDTLLLHLPELQELNSKG     60
                               ************************************************************

CsaV3_5G011180_wt              SKASIKLQPSASSGGTTWFTKSTLRRFLQIVGSPELPNIMKTMNEMSQLEETKRFHLSLY     120
CsaV3_5G011180_TGC->TGCGC      SKASIKLQPSASSGGTTWFTKSTLRRFLQIVGSPELPNIMKTMNEMSQLEETKRFHLSLY     120
                               ************************************************************

CsaV3_5G011180_wt              GQGQMSKTEEKDGCNLDSSSPKHGSGPEFASSAASKNDLLRAMDLRLTALNKDLTAAFEK     180
CsaV3_5G011180_TGC->TGCGC      GQGQMSKTEEKDGCNLDSSSPKHGSGPEFASSAASKNDLLRAMDLRLTALNKDLTAAFEK     180
                               ************************************************************

CsaV3_5G011180_wt              AHGAACSSKEISHLAKFTEHFGAINLKNCTYKYLELNPKSDNVELVNDDNKYTITSNICN     240
CsaV3_5G011180_TGC->TGCGC      AHGAACSSKEISHLAKFTEHFGAINLKNCTYKYLELNPKSDNVELVNDDNKYTITSNICN     240
                               ************************************************************

CsaV3_5G011180_wt              ENAISGSIKAEKSNSSTPVKYGVSPAKVAQIERQDSSETESSDSDNENGTPAERSRTMVR     300
CsaV3_5G011180_TGC->TGCGC      ENAISGSIKAEKSNSSTPVKYGVSPAKVAQIERQDSSETESSDSDNENGTPAERSRTMVR     300
                               ************************************************************

CsaV3_5G011180_wt              STVARRSASPMRRVQIGRTGSRRAPAIMIRSLNHLQTRDGMFSQGDAAANSDGDEEGSEP     360
CsaV3_5G011180_TGC->TGCGC      STVARRSASPMRRVQIGRTGSRRAPAIMIRSLNHLQTRDGMFSQGDAAANSDGDEEGSEP     360
                               ************************************************************

CsaV3_5G011180_wt              SGKTADNNVGRISVQDAISLFESKQKNDASDIQKRRSLANITIGANKFVLRRWSTGMGEA     420
CsaV3_5G011180_TGC->TGCGC      SGKTADNNVGRISVQDAISLFESKQKNDASDIQKRRSLANITIGANKFVLRRWSTGMGEA     420
                               ************************************************************

CsaV3_5G011180_wt              STKCHPELVSDESDPISHDLAEEVPKSKLTDEEEVGSDNISSIDKTCTTAEVEEKLEDSA     480
CsaV3_5G011180_TGC->TGCGC      STKCHPELVSDESDPISHDLAEEVPKSKLTDEEEVGSDNISSIDKTCTTAEVEEKLEDSA     480
                               ************************************************************

CsaV3_5G011180_wt              VKTSDPLETQSDSPISEPQVAVQKLSANSEWTRRKEAELDQMLKKVMESKHMAQNNSQAN     540
CsaV3_5G011180_TGC->TGCGC      VKTSDPLETQSDSPISEPQVAVQKLSANSEWTRRKEAELDQMLKKVMESKHMAQNNSQAN     540
                               ************************************************************

CsaV3_5G011180_wt              RKKDVNSEQRGELYDQYKAKRDEKRRAEEAKRNSNKEAKIKGTRQVADDRKTKIASAEVN     600
CsaV3_5G011180_TGC->TGCGC      RKKDVNSEQRGELYDQYKAKRDEKRRAEEAKRNSNKEAKIKGTRQVADDRKTKIASAEVN     600
                               ************************************************************

CsaV3_5G011180_wt              VTKKRAPRKPEVPSANLSKSEKPKK-EISKPSTIEKISSRTKPMAATRKSWPSSASERTT     659
CsaV3_5G011180_TGC->TGCGC      VTKKRAHHVSLKFHQQICQNQKNQRRKFPNH-------PRLRRFHLEQNQWLPPANPGHL     653
                               ****** :       ::.:.:* :: :: :         * : :   ::.*   *.

CsaV3_5G011180_wt              G--ISPATANATRQKAQPVSSSNRLSAKVEKSPMQKKNVKENNDSSRDLRS-------VK     710
CsaV3_5G011180_TGC->TGCGC      LHQKEPL-GFLQPQQMQHVRKPNRYPPLID-V-LKWKNLPCKRKM-RKIMIAQGT-GV-R     707
                                   .*  .    *: * * . **    ::   :: **:  :..  *.:      *   :

CsaV3_5G011180_wt              EKKEIVQAKTGKVTKTKVTLTGDSSVPVKSRIRDKVAKKSSIVPLESKSFHKGSRNSLDN     770
CsaV3_5G011180_TGC->TGCGC      KRRR-CRQRLEK-QKQKLHLLGIPPFL----SRESVT----R-PKKAA-YH---------     746
                               :::.  : :  *  * *: * *   .      *:.*:      * ::  :*

CsaV3_5G011180_wt              SSQVVSKTKPSKLSKSADSSNNSKKLTRDLEVEVTV--PDLASQPDKGDDLVPAHCDFKT     828
CsaV3_5G011180_TGC->TGCGC      ---------------------NQSPFIRVLEIA-TIVVKWLARQNL---------QNFRS     775
                                                    *.. : * **:  *:    ** *            :*::

CsaV3_5G011180_wt              VVNDQQDSEILAVDVVDADQGDVPLQQNEEKSSVEITVEGESMIPSKSTEEIEEFQELPA     888
CsaV3_5G011180_TGC->TGCGC      L-------QILQ--II-------------AKS-LVIW---------K---LRLQFLILPV     800
                               :       :**   ::              ** : *          *      :*  **.

CsaV3_5G011180_wt              NNDD---MPQLASLENTAPIENPRVR------LSLSQMLQEENSEPDSIDWGIAENPPMM     939
CsaV3_5G011180_TGC->TGCGC      NLIKGMIWCQLIAISKLL-MINRIVRYWL-M-LMLIKVMFHFNKMKRNLLWKLRLKENQ-     856
                               *  .     ** ::.:   : *  **   * ** * ::: . *.   .: * :  :

CsaV3_5G011180_wt              N--YQRGAPKGFKRLLKFARKSKGEANLAGWSSPSV------V-----------------     974
CsaV3_5G011180_TGC->TGCGC      SHLSPQRK-KSFKS---YQPIMMTCLNLHRWKTLHQLKIPVFVSLCPRCCRKKIVNLIAL     912
                               .    :   *.**    :        **  *.:         *

CsaV3_5G011180_wt              ------------SEGEDD--SEESKPLNTKKA-------DNLLMKATHNSGLVKASLDK-     1012
CsaV3_5G011180_TGC->TGCGC      IGELQKILP--TTKGVHQKDLSDS-SLQGKARGKQT-LVGQALL-FPRGR-MILKNLNL-     965
                                        ** ::* .:   .:*  *: *      *  .: *:   :.  ::  .*: *

CsaV3_5G011180_wt              NFDHEKLYSGTY------GAHNFNSKFQ-----ESHDHATVSSNKVGRSFFSLSAFRGSK     1061
CsaV3_5G011180_TGC->TGCGC      TRKRQTIY--KLLITLVL-RLHWTKILTMKSCTQVHMVHIILIPSFKK-AMIMLQFRQIK     1021
                               . .::.:*  .          ::.. :      : *    :   .. :  : :  **  *

CsaV3_5G011180_wt              ---------------      1061
CsaV3_5G011180_TGC->TGCGC      LAGLSFPYLLLGAAN      1036
```
