# Supplementary material for: Mapping Cucumber Vein Yellowing Virus Resistance in Cucumber (Cucumis sativus L.) by Using BSA-seq Analysis
Source: Front Plant Sci. 2019 Dec 3;10:1583. doi: 10.3389/fpls.2019.01583 (PMC6901629; doi:10.3389/fpls.2019.01583)
Supplement: Supplementary file 1 [file Presentation_1.zip › Supplementary figure 4.pptx]

## Slide 1
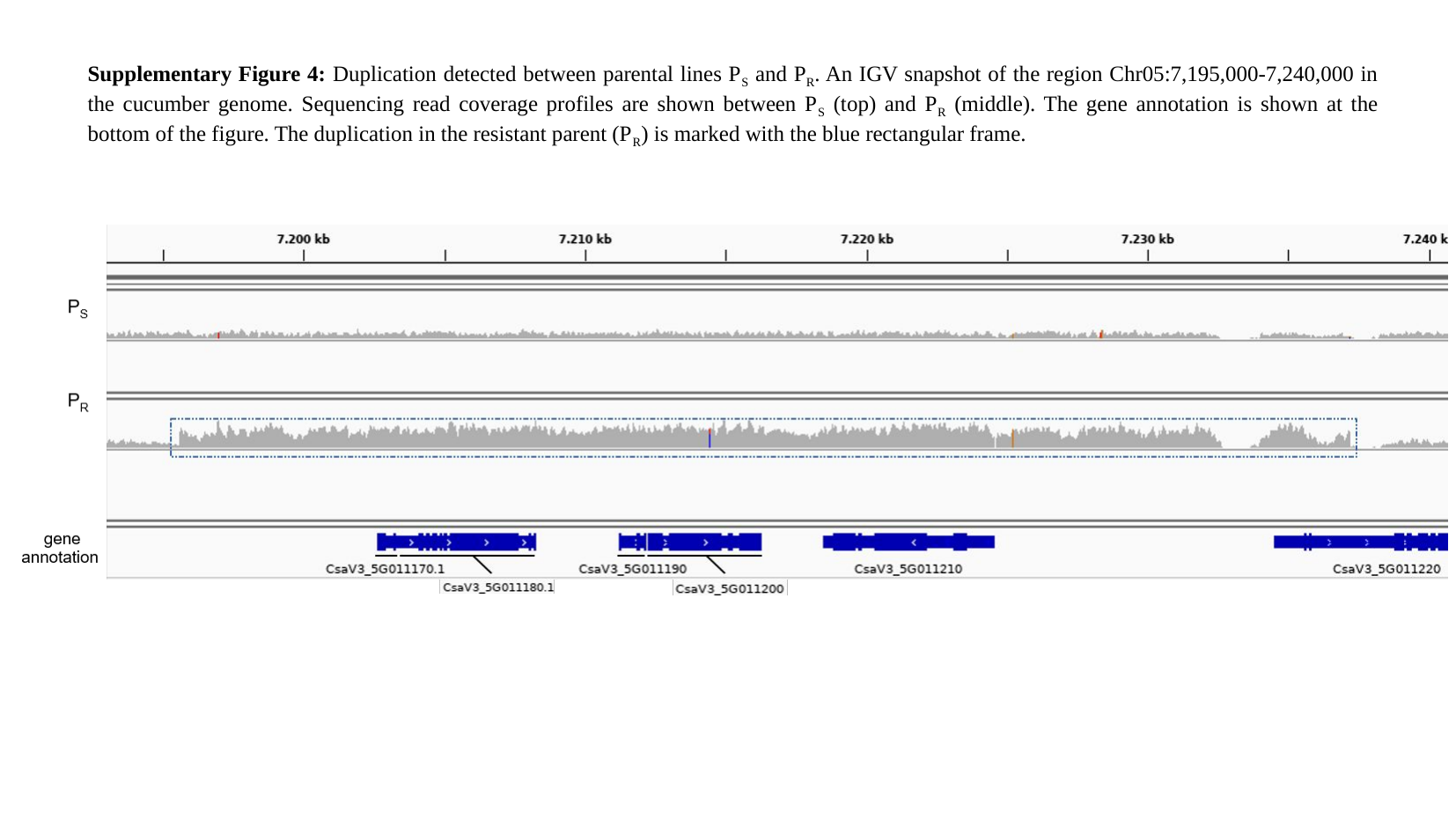

Supplementary Figure 4: Duplication detected between parental lines PS and PR. An IGV snapshot of the region Chr05:7,195,000-7,240,000 in the cucumber genome. Sequencing read coverage profiles are shown between PS (top) and PR (middle). The gene annotation is shown at the bottom of the figure. The duplication in the resistant parent (PR) is marked with the blue rectangular frame.
