# Supplementary figures and images for: Mapping Cucumber Vein Yellowing Virus Resistance in Cucumber (Cucumis sativus L.) by Using BSA-seq Analysis
Source: Front Plant Sci. 2019 Dec 3;10:1583. doi: 10.3389/fpls.2019.01583 (PMC6901629; doi:10.3389/fpls.2019.01583)

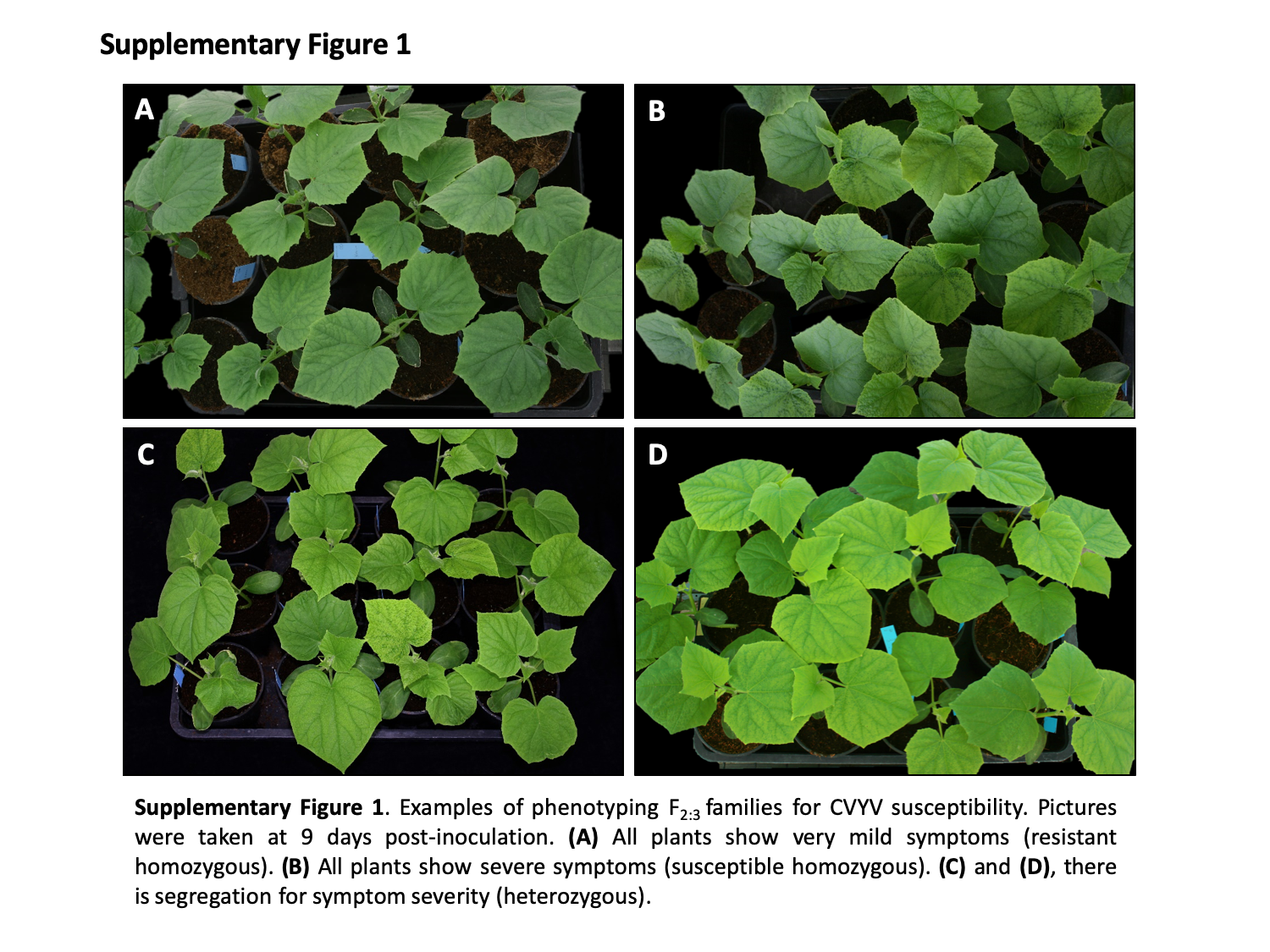

Supplement: Supplementary file 1 [file Presentation_1.zip › Supplementary figure 1.tiff]
